# Drug Reaction with Eosinophilia and Systemic Symptoms (DRESS): clinco-histopathologic studies of 45 cases

**DOI:** 10.1186/2045-7022-4-S3-P97

**Published:** 2014-07-18

**Authors:** Francois Skowron, Benoit Ben Said, Brigitte Balme, Lauriane Depaepe, D Maucort-Boulch, Jean Kanitakis, Frederic Berard, Jean Francois Nicolas

**Affiliations:** 1 CH Valence, Dermatology Department, France; 2 Chu Lyon Sud, Inserm U111, Universite Lyon 1, Clinical Immunology and Allergology Department, France; 3 Chu Lyon Sud, Pathology Department, France; 4 Hospices Civils de Lyon, Service de Biostatistiques, CNRS UMR 5558, Universite Lyon 1, Equipe Biostatistique Santé, France; 5 Chu Edouard Herriot, Pathology and Dermatology Department, France

## Background

Drug reaction with eosinophilia and systemic symptoms (DRESS) is a rare and severe adverse drug reaction. No pathognomonic test is available. The skin is the most frequent involved organ and easily sampled for a histopathological analysis. Recent series suggest that keratinocyte damage could be associated with either severe hepatic or renal involvement.

## Objectives

To study the histopathologic findings in a large homogenous series of DRESS and their link, if there is any, with visceral involvement and severity.

## Methods

We describe in a retrospective large cohort, between 2005 and 2013, 45 patients diagnosed with a DRESS (RegiSCAR's scoring system equal or more than 4) and their cutaneous histological features.

## Results

A facial edema in DRESS was associated with a definite case of DRESS (p=0.005) and a liver involvement (p=0.045). A younger age (50 years vs 66 years) was associated with a liver involvement (p=0.013). A severe neutropenia was associated with severe DRESS (p=0.012). Spongiosis and keratinocyte damage are the 2 most frequent epidermal changes noticed 55% and 53%. Pustules are not rare (26%). In the dermis a vascular alterations were frequent (88%) with a true leukocytoclastic vasculitis in 28%, a mild lymphocytic vasculitis in 13% and minor alterations in 44% of cases. In the dermis, there was a constant lymphocytic infiltrate mainly moderate in a perivascular distribution. In 57% the infiltrate is polymorphous with association of eosinophils neutrophil or atypical lymphocyte. In two third of cases, the changes are polymorphous and numerous, with no specific clear pattern. In the other one third of cases with few alterations, the presence of eosinophils, keratinocyte damage and spongiosis are usually present. A spongiosis was associated with non severe form of DRESS (p=0.041) and the absence of kidney involvement (p=0.023). A a higher of alterations per slides was associated a liver involvement (p=0.018). Our study do not support a prognostic value of keratinocyte damage.

## Conclusions

Histopathology of DRESS associated frequent spongiosis, keratinocyte damage, vascular alteration and polymorphism of the infiltrate. In two third of cases, the changes are polymorphous and numerous, in the other one third of cases with few alterations, the presence of eosinophils, keratinocyte damage and spongiosis are usually present. So cutaneous histopathology seems of little value to predict the visceral involvement.

